# Inositol Hexaphosphate Inhibits Proliferation and Induces Apoptosis of Colon Cancer Cells by Suppressing the AKT/mTOR Signaling Pathway

**DOI:** 10.3390/molecules22101657

**Published:** 2017-10-03

**Authors:** Małgorzata Kapral, Joanna Wawszczyk, Katarzyna Jesse, Monika Paul-Samojedny, Dariusz Kuśmierz, Ludmiła Węglarz

**Affiliations:** 1 Department of Biochemistry, School of Pharmacy with the Division of Laboratory Medicine in Sosnowiec, Medical University of Silesia in Katowice, Jedności 8, 41-200 Sosnowiec, Poland; jwawszczyk@sum.edu.pl (J.W.); jesse.katarzyna@gmail.com (K.J.); lweglarz@sum.edu.pl (L.W.); 2 Department of Medical Genetics, School of Pharmacy with the Division of Laboratory Medicine in Sosnowiec, Medical University of Silesia in Katowice, Jedności 8, 41-200 Sosnowiec, Poland; mpaul@sum.edu.pl; 3 Department of Cell Biology, School of Pharmacy with Division of Laboratory Medicine in Sosnowiec, Medical University of Silesia in Katowice, Jedności 8, 41-200 Sosnowiec, Poland; dkusmierz@sum.edu.pl

**Keywords:** colon cancer, InsP6, proliferation, apoptosis, AKT, mTOR

## Abstract

AKT, a serine/threonine protein kinase and mammalian target of rapamycin (mTOR) plays a critical role in the proliferation and resistance to apoptosis that are essential to the development and progression of colon cancer. Therefore, AKT/mTOR signaling pathway has been recognized as an attractive target for anticancer therapy. Inositol hexaphosphate (InsP6), a natural occurring phytochemical, has been shown to have both preventive and therapeutic effects against various cancers, however, its exact molecular mechanisms of action are not fully understood. The aim of the in vitro study was to investigate the anticancer activity of InsP6 on colon cancer with the focus on inhibiting the AKT1 kinase and p70S6K1 as mTOR effector, in relation to proliferation and apoptosis of cells. The colon cancer Caco-2 cells were cultured using standard techniques and exposed to InsP6 at different concentrations (1 mM, 2.5 mM and 5 mM). Cellular proliferative activity was monitored by 5-bromo-2′-deoxyuridine (BrdU) incorporation into cellular DNA. Flow cytometric analysis was performed for cell cycle progression and apoptosis studies. Real-time RT-qPCR was used to validate mRNA levels of *CDNK1A*, *CDNK1B*, *CASP3*, *CASP9*, *AKT1* and *S6K1* genes. The concentration of p21 protein as well as the activities of caspase 3, AKT1 and p70S6K1 were determined by the ELISA method. The results revealed that IP6 inhibited proliferation and stimulated apoptosis of colon cancer cells. This effect was mediated by an increase in the expression of genes encoding p21, p27, caspase 3, caspase 9 as well a decrease in transcription of AKT1 and S6K1. InsP6 suppressed phosphorylation of AKT1 and p70S6K1, downstream effector of mTOR. Based on these studies it may be concluded that InsP6 can reduce proliferation and induce apoptosis through inhibition of the AKT/mTOR pathway and mTOR effector followed by modulation of the expression and activity of several key components of these pathways in colon cancer cells.

## 1. Introduction

Colorectal cancer (CRC) is one of the most commonly diagnosed cancers and the third leading cause of cancer mortality in the Western countries [1,2]. The published data indicated that only 5–10% of all cancer cases are caused by genetic defects and the remaining 90–95% can primarily be ascribed to environmental factors, among which bad dietary habits are likely the most important [3]. Despite the improved diagnostic techniques and many screening programs, morbidity and mortality of CRC is still considerable [4]. Cancer prevention strongly indicates the importance of a diet and lifestyle approaches to reduce cancer risk. Among cancers, CRC is a good candidate for chemoprevention owing to the long precancerous stage before adenomas develop into cancer [5,6]. Because a diet rich in fruits, vegetables and grains is generally recognized as preventive with regard to the development of CRC, the dietary compounds responsible for this biologic effect are worth identifying. Studies on so-called nutraceutical foods have demonstrated the importance of natural compounds derived from plants in contributing to lower incidence of cancer including CRC [7,8].

One of the promising phytochemicals is inositol hexaphosphate (InsP6, IP6) which is abundant in the regular human diet, particularly in cereals, legumes, oil seeds and wheat bran, as a major fiber-associated component [9]. It is a derivative of myo-inositol, a six-fold alcohol (polyol) of cyclohexane [10], of which all carbon atoms are bonded to phosphate groups [11]. InsP6 is well absorbed from the gastrointestinal tract and cellularly internalized through pinocytosis and endocytosis [12,13]. In cells, it is partially dephosphorylated to its lower phosphorylated forms (InsP1-5) which enter the pool of inositol phosphates and act as important factors regulating diverse cellular functions [11]. Anti-cancer function of InsP6 has been demonstrated both in vivo and in vitro [14,15,16]. Its anti-cancer actions involve antioxidant properties, anti-inflammatory and immune-enhancing activities, reducing cell proliferation and inducing differentiation of malignant cells [11,17,18,19,20]. Based on this, InsP6 could be a good candidate for cancer prevention and cancer therapy. However, the understanding of the molecular mechanisms responsible for its anti-proliferative and anti-tumor activity is still limited. InsP6 has been shown to inhibit cell transformation by targeting phosphatidylinositol-3-kinase (PI3K) in JB6 mouse cells [21] and block tumor cell growth by inhibiting AKT/NF-κB-mediated survival pathway in HeLa cells [22].

The phosphatidylinositol 3-kinase/AKT/mammalian target of the rapamycin (PI3K/Akt/mTOR) signaling pathway plays a crucial role in the cell growth, proliferation and apoptosis and its dysregulations are common in a significant fraction of human malignancies, including colorectal tumors [23,24]. The serine/threonine protein kinase (AKT), also known as protein kinase B (PKB), mediates many of the downstream effects of PI3K and consequently plays central role in both normal and pathologic signaling by the PI3K pathway. The AKT family comprises three members: AKT1, AKT2 and AKT3 encoded by three different genes. All three isoforms are structurally homologous and share similar mechanisms of activation [25,26]. AKT1 and AKT2 are ubiquitously expressed in cells of many organs and AKT3 is abundant in nervous tissue [27] In turn, activated AKT (i.e., p-AKT) can phosphorylate a number of signaling proteins including caspase-9, BAD, p27^Kip1^, p21^Cip1^, glycogen synthase kinase 3α/β (GSK3). The AKT substrates play key roles in the regulation of cell cycle progression, differentiation, and survival [25,26]. Furthermore, mTOR is a known important effector of PI3K/AKT. mTOR acts as a serine/threonine protein kinase and exists in two, distinct functionally complexes, as mTORC1 and mTORC2. mTORC1 phosphorylates and activates p70S6K ribosomal kinase (S6K) and the eukaryotic translation initiation factor 4E (eIF4E) binding protein (4E-BP1), which controls protein synthesis [28,29]. In mammals, the S6K family is composed of p70S6K1, p85S6K1, p54S6K2 and p56S6K2 proteins encoded by two different genes, i.e., *S6K1* (*RPS6KB1*) and *S6K2* (*RPS6KB2*), respectively [30]. It is considered that mTORC1–S6K1 axis controls fundamental cellular processes, including transcription, translation, protein and lipid synthesis, cell growth/size and cell metabolism [29].

The clinical data revealed that AKT is aberrantly activated and p-p70S6K and p mTOR are overexpressed in human colorectal carcinoma [24,31,32]. Therefore, the AKT/mTOR signaling pathway has been recognized as an attractive target for anticancer therapy. The aim of the current study was to investigate the anticancer activity of InsP6 on colon cancer with focusing on inhibiting the AKT1 kinase and p70S6K1 as mTOR effector, in relation to proliferation and apoptosis of cells.

## 2. Results

### 2.1. Antiproliferative Activity of InsP6 on Caco-2 Cells

**Figure 1:**
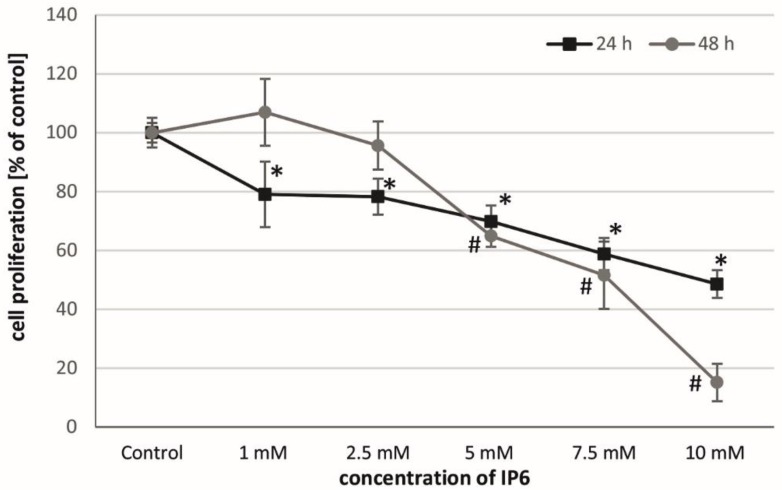
Proliferation cell inhibitory effect of InsP6 at various concentrations on Caco-2 cells after 24 and 48 h treatment. The results are expressed as percentage of untreated control (the means ± SD; * *p* < 0.05 vs. control).

The BrdU incorporation assay was used as a sensitive tool to monitor the cellular proliferative activity in response to InsP6 treatment of Caco-2 cells. As shown in Figure 1, InsP6 inhibited their DNA synthesis in a time and concentration-dependent pattern. After 24 h, InsP6 at all concentrations (1–10 mM) significantly reduced cell proliferation. Quantitative analysis of BrdU positive nuclei showed that long term (48 h) exposure of Caco-2 cells to InsP6 at a concentration of at least 5 mM reduced the level of DNA synthesis as compared to control. No relevant inhibition of cell proliferation was seen at lower concentrations up to 2.5 mM of InsP6.

### 2.2. The Influence of InsP6 on Caco-2 Cell Cycle Distribution

Flow-cytometric cell cycle analysis indicated the increase of the sub-G1 fraction of Caco-2 cells treated with InsP6. Above 3-fold higher percentage of this population was found out in the cultures treated with 5 mM InsP6 than both 1 mM and 2.5 mM IP6. About 60% cells in G1/G0 phase was detected in the control and the cultures exposed to InsP6 at lower concentrations (1 mM and 2.5 mM), however, 5 mM InsP6 markedly diminished the amount of G1/G0 cells.

**Figure 2:**
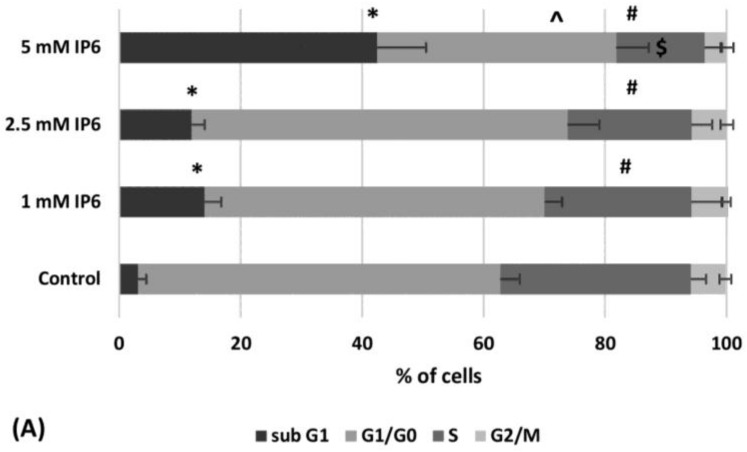

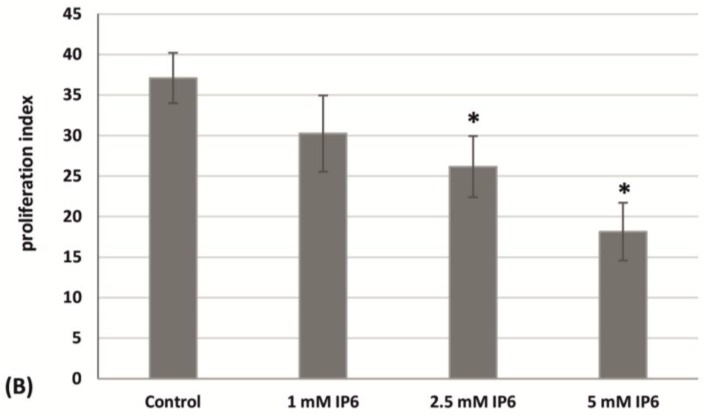
(**A**) The cell cycle distribution of the control and Caco-2 cells treated with 1 mM, 2.5 mM and 5 mM InsP6 for 48 h (the means ± SD; * (sub-G1), ^ (G1/G0), # (S phase), $ (G2/M) *p* < 0.05 vs. control). (**B**) Proliferation index of Caco-2 cells treated with InsP6 (the means ± SD; * *p* < 0.05 vs. control).

InsP6 caused a significant and concentration-dependent decrease in the percentage of cells in S phase in Caco-2 cells compared to the control cells (Figure 2A). A small number of cells observed in G2/M fraction was additionally decreased by 5 mM InsP6 in relation to control. Treatment of cells with InsP6 (1–5 mM) resulted in gradually decreasing proliferation index as compared to control (Figure 2B).

### 2.3. The Influence of InsP6 on Caco-2 Cells Apoptosis

**Figure 3:**
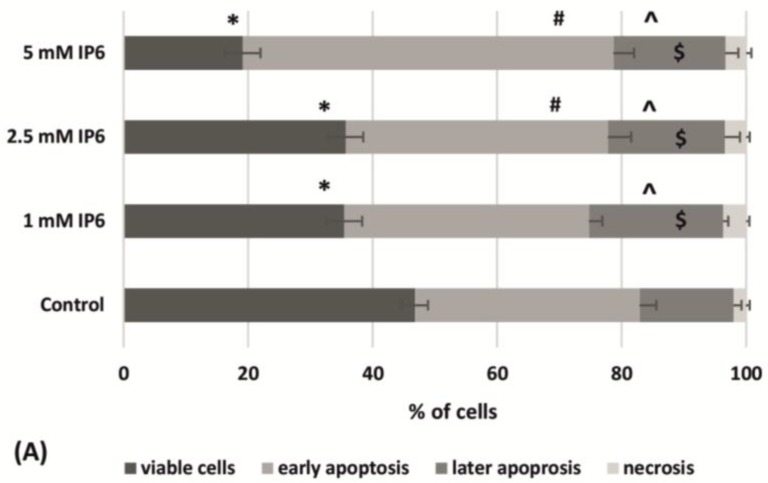

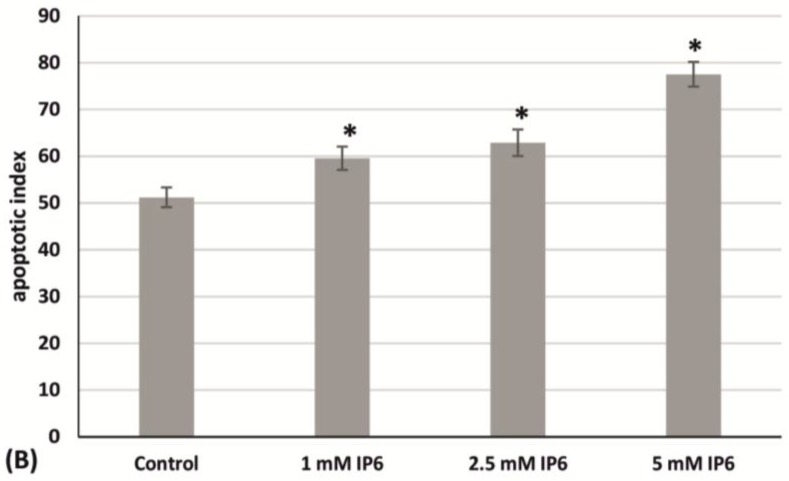
(**A**) Apoptotic and necrotic effect in the control and Caco-2 cells following treatment with 1 mM, 2.5 mM and 5 mM InsP6 for 48 h (the means ± SD; * (viable cells), # (early apoptosis), ^ (later apoptosis), $ (necrosis) *p* < 0.05 vs. control); (**B**) Apoptotic index of cells (the means ± SD; * *p* < 0.05 vs. control).

Flow-cytometric analysis indicated that InsP6 reduced the number of viable cells and increased those in early apoptosis and stronger effects were observed in cultures treated with 5 mM InsP6. The exposure of Caco-2 to InsP6 at all used concentrations revealed the presence of about 20% of cells in later apoptosis and 3.5% of necrotic cells. Statistical analysis showed that these quantities were significantly higher compared to control (Figure 3A). The obtained results indicated that InsP6 increased apoptotic index progressively to its concentration (Figure 3B).

### 2.4. The Influence of InsP6 on Transcriptional Activity of Genes Encoding p21^Waf1/Cip1^, p27^Kip1^, Caspase 3, Caspase 9, AKT1, S6K1 in Caco-2 Cells

Quantitative RT-PCR to evaluate the effect of InsP6 (1, 2.5 and 5 mM) on cell proliferation and apoptosis in colon cancer cells was performed. Caco-2 cells were incubated with InsP6 for 12 and 24 h. Next, the transcription level of *CDNK1A* and *CDNK1B* genes encoding key proteins of cell cycle, i.e., p21^Waf1/Cip1^ and p27^Kip1^, respectively was examined. The expression of *caspase 3* (*CASP3*) and *caspase 9* (*CASP9*) genes in relation to apoptotic pathways has been analyzed. Moreover, the changes of *AKT1* and *S6K1* genes expression were determined.

**Figure 4:**
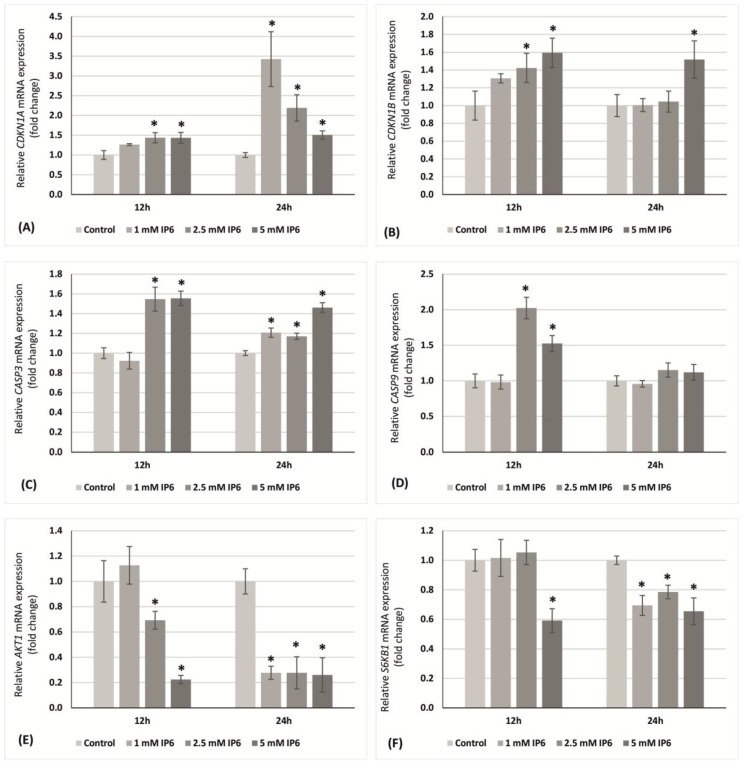
Expression of (**A**) *CDNK1A*; (**B**) *CDNK1B*; (**C**) *CASP3*; (**D**) *CASP9*; (**E**) *AKT1* and (**F**) *S6K1* genes in Caco-2 cells as determined by RT-PCR. Changes in mRNAs expression in Caco-2 cells after treatment with 1 mM, 2.5 mM and 5 mM InsP6 for 12 h and 24 h. The results are presented as mean ± SD of three separate experiments; * *p* < 0.05 vs. control.

At 12 h, 1 mM InsP6 had no effect on the transcription of both *CDNK1A* and *CDNK1B*, however, the higher concentrations of this compound significantly up-regulated genes expression in relation to control. At longer time period (24 h), InsP6 in concentration-dependent manner increased transcriptional activity of gene encoding p21, whereas a significant increase in the level of *CDNK1B* mRNA was observed with 5 mM InsP6 only (Figure 4A,B).

Compared to the control, the expression of *CASP3* and *CASP9* mRNAs in Caco-2 cells showed similar level after 12 h of 1 mM InsP6 treatment, however, InsP6 at higher concentrations significantly increased transcriptional activity of both genes. At 24 h, InsP6 in a concentration-independent manner markedly induced *CASP3* mRNA level than in control cells, while it had no effect on *CASP9* expression (Figure 4C,D).

InsP6 at lower concentration (1 mM) had no influence on *AKT1* expression at 12 h. On the contrary, the treatment of cells with InsP6 at both 2.5 mM and 5 mM decreased the amount of *AKT1* mRNA in comparison to control and 5 mM InsP6 evoked stronger effect than 2.5 mM InsP6. At 24 h, InsP6 at a concentration-independent manner significantly down-regulated the expression of this gene (Figure 4E). After 12 h, a decrease in transcription of *S6K1* gene was observed only in cultures treated with 5 mM InsP6, but longer incubation time up to 24 h resulted in significantly reduced expression of this genes in cells exposed to InsP6 at all concentrations (Figure 4F).

### 2.5. The Influence of InsP6 on the p21^Waf1/Cip1^ Protein Level in Caco-2 Cells

**Figure 5:**
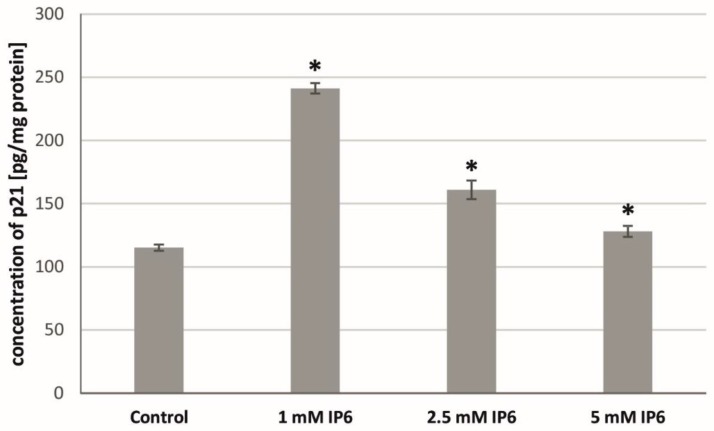
Effect of InsP6 at concentrations of 1 mM, 2.5 mM and 5 mM on the p21^Waf1/Cip1^ concentration in Caco-2 cells at 24 h. The results are presented as mean ± SD of three separate experiments; * *p* < 0.05 vs. control.

At the next step of the study, the effect of InsP6 on the concentration of p21^Waf1/Cip1^ protein in Caco-2 cells has been evaluated. The exposure of the cells to InsP6 at all concentrations for 24 h resulted in an up-expression of p21 protein as compared with untreated cells (Figure 5). The obtained results revealed the higher amount of this protein in cultures treated with 1 mM InsP6 than those treated with compound at the concentrations of 2.5 mM and 5 mM.

### 2.6. The Influence of InsP6 on Caspase-3 Activity in Caco-2 Cells

**Figure 6:**
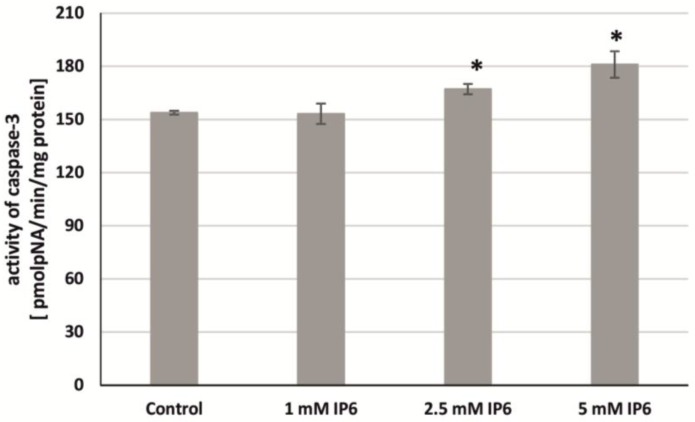
Effect of InsP6 at concentrations of 1 mM, 2.5 mM and 5 mM on the caspase-3 activity in Caco-2 cells at 24 h. The results are presented as mean ± SD of three separate experiments; * *p* < 0.05 vs. control.

In order to confirm the early stages of apoptosis, the impact of InsP6 on caspase-3 activity in colon cancer cells was estimated. The analysis showed that the enzyme activity was induced by InsP6 in a concentration-dependent manner in Caco-2 compared with the untreated cells. As shown in Figure 6, 24 h exposure of cells to InsP6 at higher concentrations (2.5 and 5 mM) resulted in significant increases in caspase-3 activity in relation to control. In the cells incubated with 1 mM InsP6 caspase activity did not alter when compared to untreated cells.

### 2.7. The Influence of InsP6 on AKT1 Activity in Caco-2 Cells

**Figure 7:**
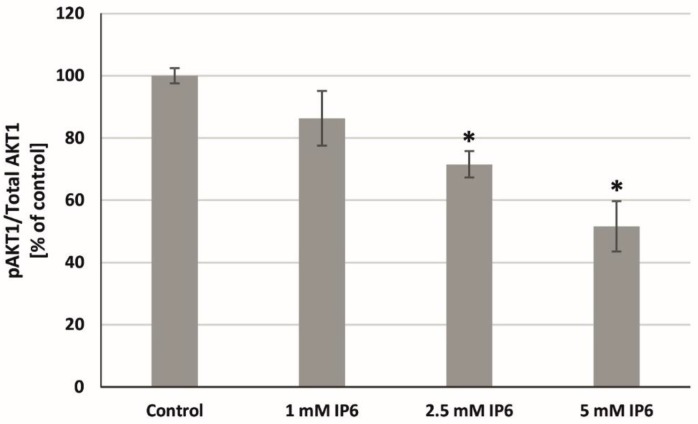
Effect of InsP6 at concentrations of 1, 2.5 and 5 mM on the AKT1 activity in Caco-2 cells at 24 h. The results are presented as mean ± SD of three separate experiments; * *p* < 0.05 vs. control.

Taking into consideration that AKT1 might play a critical role in external stimuli-induced signaling pathways, the effect of InsP6 on AKT1 phosphorylation in Caco-2 cells has been tested. As shown in Figure 7, serine 473 AKT1 phosphorylation was significantly inhibited by 2.5 mM and 5 mM InsP6 in relation to control. Much stronger p-AKT suppression was observed in cells treated with 5 mM InsP6. InsP6 at the lowest concentration reduced this process, however, comparative analysis revealed no markedly difference.

### 2.8. The Influence of InsP6 on p70S6K Activity in Caco-2 Cells

**Figure 8:**
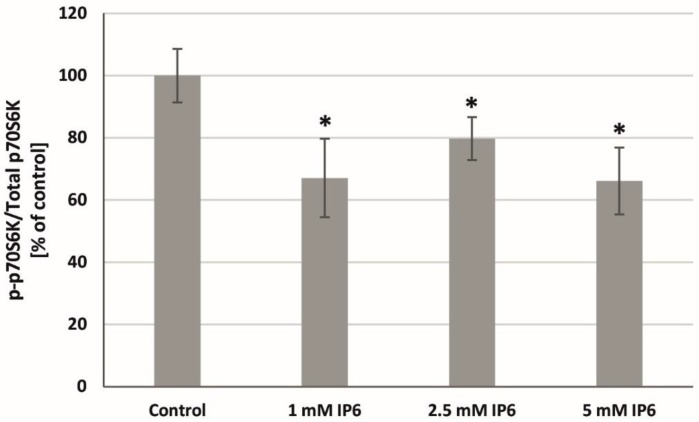
Effect of InsP6 at concentrations of 1 mM, 2.5 mM and 5 mM on the p70S6K activity in Caco-2 cells at 24 h. The results are presented as mean ± SD of three separate experiments; * *p* < 0.05 vs. control.

To evaluate the effect of InsP6 on mTOR activity, a p-p70S6K(Thr389)/total p70S6K ratio was determined. mTORC1 phosphorylates and activates S6K1, therefore, phosphorylated S6K1 is considered as biomarker of mTOR activation. The obtained results showed that InsP6 in dose-independent manner significantly reduced the activity of p70S6K in Caco-2 compared with control (Figure 8).

## 3. Discussion

**Figure 9:**
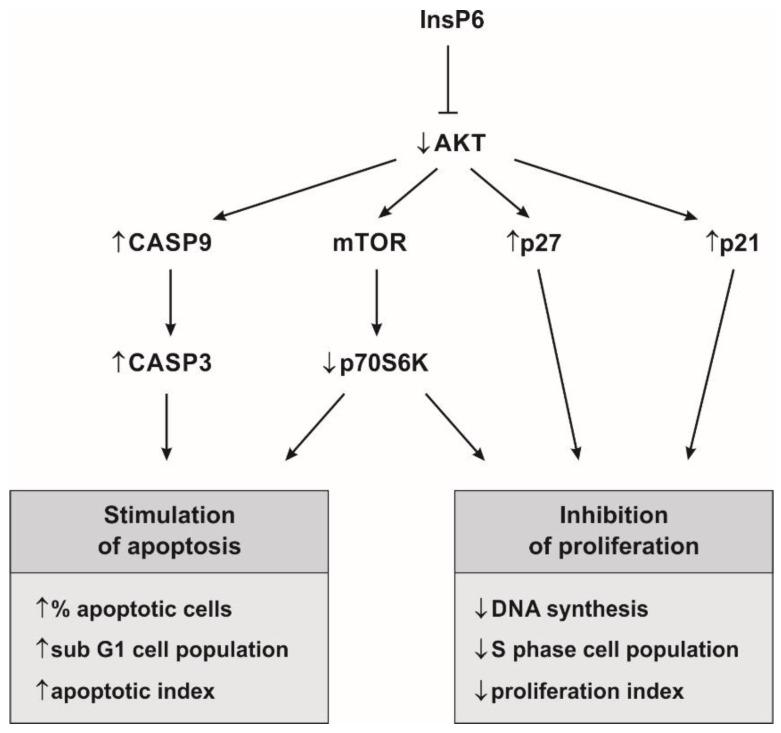
Scheme of InsP6 molecular pathways and targets in anti-cancer activities.

In the present study, we demonstrated the suppressive effect of InsP6, a natural occurring phytochemical, on the expression and activity of key components of the AKT/mTOR signaling axis including AKT1 and p70S6K1 in colon cancer cells as significant regulators of proliferation and apoptosis (Figure 9). Cellular death and proliferation occur through tightly regulated processes. Cancer cell survival reflects an imbalance between its proliferation and apoptosis. Therefore, the main goal of strategy of cancer treatment and prevention is to inhibit the growth and proliferation of tumor cells and to promote their death [33]. PI3K/AKT is the most targeted pathway in human cancers, since its activation leads to cell proliferation and cell survival [34]. AKT kinase is an important molecular junction in intracellular cell signaling and its activation is considered as both necessary and sufficient for cell survival. In response to PI3K activation this enzyme phosphorylates and regulates the activity of a number of targets, including kinases, transcription factors and other regulatory molecules [26]. In particular, it has been demonstrated that AKT promotes cell survival through phosphorylation and inactivation of several proapoptotic proteins, including caspase-9 which participates in intrinsic pathway of apoptosis linked to mitochondrial functions. During apoptosis, caspase 9 activates pivotal effector caspase 3. The activation and cleavage of caspase 3 is a marker of the execution phase, the final phase of apoptosis [35,36]. Furthermore, AKT activation affects cell cycle progression through regulation of cyclins stability and inhibition of the cyclin-dependent kinase inhibitors such as CDKN1A (p21) and CDKN1B (p27) level [27]. Both p21 and p27 regulate the progression of cell cycle in the G0/G1 phase, and overexpression of these proteins blocks the G1 to S transition [37]. Besides, AKT activates the transcription factors NF-κB and FOXO (Forkhead family of transcription factors) which results in transcription of anti-apoptotic genes as well *CDKN1B* gene [25,26]. These data point to AKT as a promising target for therapeutic purpose inhibition, as blocking AKT may have direct effects on both apoptosis and proliferation processes. Additionally, AKT via mTOR pathway indirectly regulates cell growth and proliferation and protein synthesis by phosphorylating p70S6K which subsequently phosphorylates the ribosomal protein S6 leading to enhanced translation [30,38].

AKT/mTOR is an attractive target for the development of novel inhibitors that might prove beneficial in the treatment of cancers in which this pathway is constitutively activated [24,27]. Conventional therapies for cancer are usually associated with numerous side effects for patients. In recent years, growing attention has been focused on natural molecules that represent a promising group of anticancer agents due to their multiple targets in cancer cells with limited toxic effect on normal cells [8]. Over the three past decades, numerous studies have proven anti-cancer properties of InsP6, a natural compound regularly consumed in foods containing cereals and legumes, in some cancer, such as prostate [14,39,40,41], pancreatic [42], breast [15,43], malignant melanoma [44] and colon [19,20,33,43,45,46] in animal models or in vitro studies. These papers have reported that InsP6 reduced growth and proliferation cells in multiple cancers. InsP6 has been shown to exert its inhibitory effect by G0/G1 phase arrest and S phase restriction which are associated with modulation of cell-cycle regulatory proteins such as p53, pRb, cyclins, cyclin-dependent kinases and their inhibitors [15,20,39,40,41]. Moreover, InsP6 induced apoptosis through regulating pro- and apoptotic proteins (Bax, Bcl-xl) and increasing the expression and activation of caspases (CASP3, CASP8, CASP9) [19,33,39,46]. On the other hand, in the normal cells InsP6 seems to exert an opposite activity, i.e., an anti-apoptotic function. The result of Verbsky and Majerus [47] showed that it protected normal embryonic kidney HEK293 cells from apoptosis induced by TNFα as well mediated by Fas.

In the current study, we investigated whether InsP6 inhibits AKT/mTOR signaling cascade in Caco-2 colon cancer cells as a significant regulator of proliferation and apoptosis. The results of this experiment remain in agreement with the previously published in vivo [14,48] and in vitro [15,19,39] researches which also showed inhibitory effect of InsP6 on the expression and activity of AKT kinase. InsP6 decreased in a concentration-dependent manner the level of p-AKT1/total AKT1 and transcriptional activity of gene encoding this isoform following Caco-2 cells treatment for 12 h. Prolonged action of InsP6 regardless of its concentration evoked down-expression of the *AKT1* gene. InsP6 down-regulation of *AKT* expression may contribute to the reduction of AKT activity. In this study, for the first time, we investigated the influence of InsP6 on the activity of mTOR by means of p70S6K1 activity analysis in human colon cancer cells. It has well been established that S6K activation absolutely required TORC1-mediated phosphorylation [38] and phosphorylated S6K1 has been recognized as biomarker of mTOR activation [49]. The obtained results revealed that InsP6 at the highest concentration down-regulated transcriptional activity of *S6K1* gene at 12 h. However, the prolonged incubation time of cells with InsP6 resulted in a concentration-independent manner decrease in the mRNA expression of *S6K1* and reduced activity of p70S6K. In consequence, InsP6 inhibited proliferation of colon cancer cells and the inhibition correlated with the up-regulation of the mRNA expression of *CDNK1A* and *CDNK1B* genes. Furthermore, InsP6 increased the level of p21 protein, cyclin-dependent kinase inhibitor in Caco-2 cells. Our previously published studies also revealed InsP6 stimulated down-regulation of the expression of cyclin D1 in colon cancer cells [50]. Concomitantly, InsP6 induced apoptosis of colon cancer cells which was accompanied by upregulated expression of caspase 9 and caspase 3 at transcriptional level as well increase in caspase-3 activity (Figure 9). The proapoptotic effect of InsP6 was strongly manifested in cultures treated with its higher concentrations. In the present study Caco-2 cells exposed to 5 mM InsP6 have undergone apoptosis as evidenced by a significant increase in the sub G1 phase cell population and early apoptotic cells accumulation as well as marked decrease in the amount of S phase cells. Moreover, these observations have been confirmed by augmented activity of executioner caspase 3 and enhanced expression of its gene. In turn, InsP6 at physiological concentration (1 mM) suppressed cell growth as manifested by G0/G1 phase cells accumulation and the highest up-expression of *CDNK1A* gene at both mRNA and protein levels. InsP6 at concentration of 2.5 mM reduced proliferation and stimulated apoptosis in Caco-2 cells which was reflected by the significant quantitative changes of markers of these processes. According to Singh et al. [39], the endogenous cellular/physiological levels of InsP6 (100 μM–1 mM) were not sufficient for its anti-cancer activity in cell cultures, and higher levels of InsP6 were likely required for its pharmacological efficacy. The results of our study confirm the previous published data that InsP6 exerted most biological effects at concentration of 2 mM in cell cycle studies and at 4 mM in both cell cycle and apoptosis studies [39]. These concentrations of IP6 correspond to its concentrations in the lumen, reaching on average 4 mM [51]. Taken together, the present results indicate that InsP6 can inhibit cell proliferation and induce apoptosis through downregulating AKT/mTOR signaling pathway.

Despite many years of research, the molecular mechanism of the antitumor effect of InsP6 is still unclear. Various theories have been proposed for elucidating the anticancer activity of this nutraceutical. One of them shows that InsP6 through acting on PI3K/AKT signal transduction pathway can influence cell cycle regulation, growth, and differentiation of malignant cells [14,19]. According to Huang et al. [21] InsP6 may inhibit PI3K due to the similarity of its structure to d-3-deoxy-3-fluorophosphatidylinositol, a potent PI3K inhibitor. On the other hand, InsP6 is known to be a strong metal ion chelator, and it may inhibit the activity of kinases by chelating divalent cations, affecting PI3K/AKT pathway [52]. It is also speculated that the anticancer effect of InsP6 is mediated through its conversion to lower inositol phosphates which play crucial roles in cellular signal transduction [11,22]. The published data demonstrated that InsP5 inhibited AKT phosphorylation and activity and promoted apoptosis via PI3K/AKT pathway in small cell lung cancer (SCLC-H69), ovarian cancer (SKOV3) and breast cancer (SKBR-3) cell lines [53]. Maffucci et al. [54] observed that InsP6 did not affect AKT phosphorylation when tested in short-time experiments, whereas it inhibited AKT at longer incubations. The observation of such an effect only at longer incubation strongly suggests that it is likely due to a conversion of inositol polyphosphate to its different forms, likely InsP5. Additionally, it has been shown that the myo-inositol, a InsP6 precursor, also reduced PI3K expression and AKT activity [55]. Therefore, it is possible that InsP6 could exert its effects through myo-inositol.

AKT/mTOR pathway participates in the regulation of carbohydrate and lipid metabolism. AKT induces glucose uptake by increasing the expression of glucose transporters (GLUT1, GLUT4), and promoting GLUT4 translocation to the plasma membrane. It also phosphorylates and activates phosphofructokinase-2 (PFK2) which allosterically activates phosphofructokinase-1 (PFK1) the rate-limiting enzyme of glycolysis. Furthermore, AKT via inhibition of glycogen synthase kinase 3 (GSK3) decreases glycogen synthesis and regulates lipid metabolism. AKT has been shown to phosphorylate and activate ATP citrate lyase which provides acetyl-Co, i.e., a precursor of fatty acids and cholesterol. On the other hand, inhibition of AKT enhances β-oxidation of fatty acids [56,57]. Furthermore, mTORC1 increases de novo lipid synthesis by activating sterol regulatory element-binding protein (SREBP), a transcription factor for lipogenic genes [58]. The published data indicated that AKT/mTOR deregulation occurs in human metabolic diseases, such as type 2 diabetes or obesity [59,60]. Because of the ability of InsP6 to inactivate key components of this pathway it may be considered as a useful agent supporting the treatment of metabolic diseases.

Based on the present studies it may be concluded that InsP6 can reduce proliferation and induce apoptosis through inhibition of AKT/mTOR pathway and mTOR effector followed by modulation of the expression and activity of several key components of this pathway in colon cancer cells. These results may provide new insights into the interventional strategies against colon cancers. Furthermore, it should be mentioned that the PI3K/AKT and mTOR/p70S6K pathways are main signaling pathways that negatively regulate autophagy, other type of programmed cell death [61], so further studies are required in order to confirm whether InsP6 induces simultaneously apoptosis and autophagy. Our data suggest potential usefulness of InsP6 as a novel therapeutic modulator of AKT/mTOR signaling cascade, an important prognostic factor in human colorectal cancer. More studies are still needed to elucidate the protective functions of InsP6 and its utilization in the prevention and therapy of cancer.

## 4. Materials and Methods

### 4.1. Cell Culture

Human colon cancer Caco-2 cell line was obtained from the American Type Culture Collection (ATCC, Rockville, MD, USA). Cells were grown in RPMI 1640 medium (Sigma Aldrich, St. Louis, MO, USA), supplemented with 10% fetal bovine serum (Biowest, Nuaillé, France), 100 U/mL penicillin and 100 μg/mL streptomycin (both from Sigma Aldrich) and 10 mM HEPES (Sigma Aldrich) in a humidified atmosphere containing 5% CO_2_ and 95% air at 37 °C. For each assay, cells were seeded onto culture dishes and routinely cultured for 48 h. Cells were treated with IP6 for the desired concentrations and times and proceeded for analysis as described below. The untreated cells were used as the control.

### 4.2. Preparation of InsP6 Solution

A 250 mM stock solution of InsP6 (dipotassium salt) (Sigma Aldrich) was prepared by dissolving it in pyrogen-free water and adjusting to pH 7.4. The stock solution was then diluted with cell culture medium to achieve different concentrations of InsP6 prior to their immediate use.

### 4.3. BrdU Incorporation Assay

Cell proliferation was assessed using a bromodeoxyuridine (BrdU) ELISA kit according to manufacturer’s instruction (Roche, Mannheim, Germany). Briefly, cells were cultured at 8 × 10^3^ cells/mL in a 96-well plate and treated with InsP6 at the concentrations of 1–10 mM for 24 h and 48 h. BrdU labeling solution was added for the last 6 h of incubation. Cells were fixed and denatured for 30 min, and then incubated with anti-BrdU-POD for 90 min at room temperature. BrdU incorporation was determined by absorbance measuring at λ = 450 nm (with reference λ = 690 nm) using a microplate spectrophotometer (Labtech International Ltd., Uckfield, UK).

### 4.4. Cell Cycle Analysis

Caco-2 cells (2.2 × 10^5^ cells) were seeded onto 21.5 cm^2^ dishes (Nunc International, Rochester, NY, USA) and treated with InsP6 at the concentrations of 1 mM, 2.5 mM and 5 mM for 48h. They were then harvested by trypsinization, washed in PBS buffer and fixed in cold 70% ethanol at −20 °C overnight. The cell pellets were incubated with RNaseA (final concentration, 200 μg/mL) in PBS buffer for 1 h at 37 °C in the dark and then stained with propidium iodide solution (Sigma Aldrich, final concentration, 10 μg/mL). The DNA content and cell cycle distribution of the cells were analyzed by BD FACS Aria II flow cytometer and BD FACSDiva software (BD Biosciences, San Jose, CA, USA). The proliferation index (PI), i.e., percentage of proliferating cells in the S + G2/M cell cycle phases was determined.

### 4.5. Apoptosis Assay

Caco-2 cells (2.2 × 10^5^) were seeded onto 21.5 cm^2^ dishes (Nunc International) and then exposed to InsP6 at the concentrations of 1 mM, 2.5 mM and 5 mM for 48 h. Floating and adherent cells were harvested by trypsinization, washed twice with Hank’s Balanced Salt Solution (HBSS, Sigma Aldrich). Pelleted cells were resuspended in 1 mL of HBSS and then stained with the use of Vybrant Dye Cycle Violet/SYTOX AADvanced Apoptosis Kit (Invitrogen, Carlsbad, CA, USA) as described in the manufacturer’s protocol. Viable and dead cells were detected by flow cytometry using BD FACS Aria II detector. The analysis of stained cells allowed to differentiate into four groups, i.e., viable, early apoptotic, late apoptotic and necrotic cells. The apoptotic index, i.e., percentage of early and late apoptotic cells was determined.

### 4.6. Total RNA Extraction and Quantitative Real-Time RT-PCR (RT-qPCR)

To evaluate transcriptional activity of *CDNK1A*, *CDNK1B*, *CASP3*, *CASP9*, *AKT1*, and *S6K1* genes, the cells were seeded at a density of 8 × 10^5^ onto 21.5 cm^2^ culture dishes (Nunc International). Then, InsP6 at the concentrations of 1 mM, 2.5 mM and 5 mM was added to cell cultures for 12 h and 24 h. Total RNA was extracted from the cells with the use of TRI REAGENT (Zymo Research, Irvine, CA, USA) according to manufacturer’s instructions. RNA concentration and purity were checked using the Shimadzu UV-1800 spectrophotometer (Shimadzu, Kyoto, Japan). Samples showing a ratio of Abs 260/280 nm between 1.8 and 2.0 were only used for experiments. Detection of the expression of examined genes was carried out using a RT-qPCR technique with a SYBR Green chemistry (SYBR Green Quantitect RT-PCR Kit) (Qiagen Inc., Valencia, CA, USA) and CFX Connect Real-Time PCR Detection System (Bio-Rad, Hercules, CA, USA). Aliquots (0.1 μg) of total cellular RNA were applied to one-step RT-qPCR in a 20 μL reaction volume. Oligonucleotide primers specific for *CASP3* and *CASP9* mRNAs were synthesized in Oligo.pl at the Institute of Biochemistry and Biophysics of the Polish Academy of Sciences (Warsaw, Poland). Primers for *CDNK1A*, *CDNK1B*, *AKT1* and *S6K1* were commercially available (Sigma Aldrich). Characteristics of primers are presented in Table 1. The thermal profile for RT-qPCR was as follows: 50 °C for 30 min for reverse transcription and 95 °C for 15 min followed by 45 cycles at 94 °C for 15 s, 55 °C for 30 s and 72 °C for 45 s for amplification. Each gene analysis was performed in triplicate. The mRNA copy numbers of examined genes were determined on the basis of the commercially available standard of β-actin (TaqMan DNA Template Reagent Kit, Invitrogen) and recalculated per μg of total RNA. The expression levels of all genes in cultured cells were expressed as the fold change relative to the corresponding controls. A values of fold change >1 and <1 were set up as an increased and a decreased expression of the target gene, respectively.

### 4.7. Measurement of the p21^Waf1/Cip1^ Protein Level

To determine the p21^Waf1/Cip1^ concentration, the cells were seeded at a density of 3 × 10^6^ onto 56.7 cm^2^ culture dishes and cultured with InsP6 at the concentrations of 1 mM, 2.5 mM and 5 mM for 24 h. Afterwards, cells were washed with ice-cold PBS, scrapped from the dishes and centrifuged. They were lysed on ice in cell extraction buffer. The expression of p21^Waf1/Cip1^ protein in Caco-2 cells was determined with commercially available ELISA kits (Invitrogen) following the producer’s instruction. The absorbance was measured using the multiplate reader Labtech LT-5000 (Labtech International) at λ = 450 nm. The concentration of p21 was compared with the standard curve generated under identical conditions. The values were normalized to the total protein content in cells, as measured by the Bradford assay (Sigma Aldrich).

### 4.8. Measurement of Caspase-3 Activity

Caspase-3 activity in Caco-2 cells was determined with commercially available Colorimetric Caspase 3 Assay Kit (Sigma Aldrich) according to manufacturer’s instruction. The assay is based on the hydrolysis of the peptide substrate acetyl-Asp-Glu-Val-Asp p-nitroanilide (Ac-DEVD-pNA) by caspase 3, resulting in the release of the *p*-nitroaniline (pNA) moiety. Prior to the experiments, the cells (1 × 10^6^) were incubated with InsP6 at concentrations of 1 mM, 2.5 mM and 5 mM for 24 h. Next, cells were scraped from the dishes, lysed, centrifuged and supernatants were added to reaction buffer containing caspase-3 substrate (DEVD-pNA). The absorbance of released pNA was measured using a Labtech LT-5000 multiplate reader (Labtech International) at λ = 405 nm. The specificity of the assay was confirmed by the parallel determination of the caspase-3 activity in the cell lysates in the presence of the specific enzyme inhibitor (Ac-DEVD-CHO). The caspase-3 activity in cell lysates was calculated relative to cellular protein content determined by Bradford’s method.

### 4.9. The p70S6K Activity Assay

The level of both total and phosphorylated p70 S6K (Thr389) proteins in Caco-2 cells was determined simultaneously with commercially available InstantOne ELISA kits (Affymetrix eBioscience, Carlsbad, CA, USA). Caco-2 cells were cultured at 1 × 10^4^ cells/mL in a 96-well plate and treated with InsP6 at the concentrations of 1 mM, 2.5 mM and 5 mM for 24 h. Afterwards, cells were lysed in commercial available buffer. The concentrations of total p70S6K and p-p70S6K in lysates were measured with ELISA kits according to manufacturer’s protocols. The absorbance was measured using the Labtech LT-5000 multiplate reader (Labtech International) at λ = 450 nm. The obtained results were calculated as a ratio of p-p70S6K/total p70S6K and expressed as percentage of that in control.

### 4.10. The AKT1 Activity Assay

The level of both total and phosphorylated AKT (Ser473) proteins in Caco-2 cells was determined simultaneously with commercially available ELISA kits (RayBio, Norcross, GA, USA). Caco-2 cells (7 × 10^5^) were incubated with InsP6 at the concentrations of 1 mM, 2.5 mM and 5 mM for 24 h. Then, they were lysed in cell lysate buffer and the contents of both AKT and p-AKT was measured as recommended by the producer. The absorbance was measured using the multiplate reader Labtech LT-5000 (Labtech International) at λ = 450 nm. The obtained results were calculated as a ratio of p-AKT1/total AKT1 and expressed as percentage of that of control.

### 4.11. Statistical Analysis

Statistical analysis was performed with the use of Statistica PL 12.0 software (Statistica, Tulsa, OK, USA). All data expressed as means ± SD were representative of at least three independent experiments. One-way analysis of variance (ANOVA) with Tukey’s post-hoc test was used to evaluate significances between examined groups. Values of *p* < 0.05 were considered as statistically significant.

## Figures and Tables

**Table 1:** Characteristics of the primers used in the experiments.

Gene	Primer Sequence	Product Size (Base Pair)
*CDNK1A*	F: 5′ AGGGATTTCTTCTGTTCAGG 3′	185
	R: 5′ GACAAAGTCGAAGTTCCATC 3′	
*CDNK1B*	F: 5′ AAAATGTTTCAGACGGTTCC 3′	93
	R: 5′ ATTCGAGCTGTTTACGTTTG 3′	
*CASP3*	F: 5′GGCCTGCCGTGGTACAGAACTGG 3′	174
	R: 5′ AGCGACTGGATGAACCAGGAGCCA3′	
*CASP9*	F: 5′GACCGGAAACACCCAGACCAGTGGA 3′	125
	R: 5′GCAGTGGCCACAGGGCTCCAT 3′	
*AKT1*	F: 5′AAGTACTCTTTCCAGACCC 3′	197
	R: 5′ TTCTCCAGCTTGAGGTC 3′	
*S6KB1*	F: 5′ATTCATGATGGAACAGTCAC 3′	121
	R: 5′ACATTAATGCTCCCAAACTC 3′	
